# As to Drinking

**Published:** 1910-10

**Authors:** 


### Abstracts and Selections.

### As to Drinking.

Laying aside any and all superstition that has been handed down from the early dawn of history, and resting man's standing in the universe on the absolute basis of facts and results, it does not necessarily need a scientific observer to realize that the drinking of alcohol has been one of the greatest curses to which humanity has been heir.

On the cold basis of common sense, no rational being can point to a single instance where its imbibition has been beneficial to himself, to his family, to his friends or to his country.

As time rolls on and people emerge further and further from the bonds of ignorance, superstition and custom with which the ages have bound them, so that they may see things in the clear light of reason and common sense, the great wonder will be as to the amount of money wasted in alcohol the diseases created, the crimes committed, and the hearts broken under the influence of intoxicating liquors.

Among other sad words of tongue or pen are these: we have to unlearn many things on account of faulty teaching in early years.

Custom is one of the most difficult things in the world to transgress, and there are, comparativley speaking, few men who have the personal initiative to overcome such a barrier.

In the career of a family physician there are so many hundreds of instances of misery that are directly attributable to drinking that I often wonder how any physician can condone such use of alcohol and its derivatives.

In my early years I was so thoroughly imbued with the doctrine of personal liberty being inherent in the free-born American that I failed to recognize the fact that any personal liberty which transcends the rights of another individual, be it wife, mother, father or child, is not a liberty but a license, and as such should have no standing among civilized people.

In my earlier days I did not believe in prohibition. I do not believe today that it is a practicable measure in any community where the people are so thoroughly steeped in ignorance and selfishness and bestiality that they can not deny their bellies what their appetite craves.

Whisky drinking is not a political question. It is a moral, ethical and economic proposition which should occupy the very best thought of all sound minds.

From the cold-blooded standpoint of economics, the drinking of whisky is indefensible, as being absolutely without any other result than crime, incapacity, selfishness, and the creation of a countless number of idiots, thieves, prostitutes, sick and afflicted, and all those inept creatures which civilization has to care for.

Besides it is the most potential factor in causing municipal corruption and maladministration with which this country has to contend.

The medical profession owes it to itself and to the people at large that they join in every effort to impress on the public the utter inadvisability of drinking.—*Mississippi Medical Monthly.*

